# Epidemiology of Therapy-Related Myeloid Neoplasms After Treatment for Pediatric Acute Lymphoblastic Leukemia in the Nordic Countries.

**DOI:** 10.4084/MJHID.2011.020

**Published:** 2011-05-16

**Authors:** Kjeld Schmiegelow

**Affiliations:** Faculty of Medicine, Institute of Gynecology, Obstetrics and Pediatrics, and the Department of Pediatrics, the University Hospital Rigshospitalet, Copenhagen, Denmark

## Abstract

Of 1614 Nordic children with ALL that were treated according to the NOPHO ALL92 protocol, 20 developed an SMN (cumulative risk at 12 years: 1.6%). Sixteen of the twenty SMNs were acute myeloid leukemias or myelodysplasias, and 9 of these had either monosomy 7 (n=7) or 7q deletions (n=2). In Cox multivariate analysis longer duration of oral MTX/6MP maintenance therapy (p=0.02; being longest for standard risk patients) and presence of high-hyperdiploidy (p=0.07) were related to an increased risk of SMN. In 524 patients we determined the erythrocyte activity of thiopurine methyltransferase (TPMT), which methylates 6MP and its metabolites, and thus reduces cellular levels of cytotoxic 6-thioguanine nucleotides. The TPMT activity was significantly lower in those that did compared to those that did not develop an SMN (Median: 12.1 vs 18.1 IU/ml; p=0.02). Among 427 TPMT wild type patients, those who developed SMN received higher 6MP doses than the remaining (69.7 vs 60.4 mg/m^2^, p=0.03), which may reflect increased levels of methylated metabolites that inhibit purine *de novo* synthesis and thus enhance incorporation of 6-thioguanine nucleotides into DNA. In conclusion, the duration and intensity of 6MP/MTX maintenance therapy of childhood ALL may influence the risk of SMN.

## Introduction:

One of the most devastating complications to the therapy of childhood acute lymphoblastic leukemia (ALL) is the development of second cancers (SMN), a complication that in general has a poor prognosis.^1^–^5^ Although on average it occurs in 2% of all patients depending on the therapy given, the published cumulative incidences vary from less than 1% to almost 10%.^6^–^8^ Furthermore, since the overall survival of childhood ALL now approaches 85%, SMN now encompass 15–20% of all deaths after childhood ALL therapy.

The incidence of SMN will be influenced not only by the treatment modalities applied but also by the duration of follow-up, since SMN may emerge several decades from the initial ALL treatment. Furthermore, some centres actively screen for subclinical cancers, e.g. meningeomas^9^ and thyroid cancers.^10^ Finally, the strategies or reporting of SMNs differ widely between collaborative groups ranging from no-news-are-good-news to follow-up by regular questionnaires or systematic exchange of data with national or regional cancer registries, which in the Nordic countries are population-based and enforced by legislation and by exchange of data with the National hospital discharge registration.

Treatment-related risk factor analyses have primarily focused on irradiation, alkylating agents, and topoisomerase II inhibitors (anthracyclines, epipodophyllotoxins) that induce DNA damage, whereas less attention has been paid to mechanisms that interfere with DNA control, including drugs that may modify DNA repair.^5^ Previously, children with higher risk ALL had the highest risk of SMN due to their more intensive chemotherapy and radiotherapy and the use of hematopoietic stem cell transplantation. However, studies from the Nordic Society for Paediatric Haematology and Oncology (NOPHO) and from St Jude Children’s Research Hospital have indicated that even the less intensive 6-mercaptopurine (6MP)/methotrexate (MTX) maintenance therapy may enhance the risk of SMN not least for patients with thiopurine methyltransferase (TPMT) low-activity polymorphisms.^11^,^12^ Since TPMT methylates 6MP and some of its metabolites and thus reduces the intracellular amounts of cytotoxic 6-thioguanine nucleotides (6TGN) available for DNA incorporation, the 5–10% of patients who have low TPMT activity will have higher intracellular 6TGN levels for DNA incorporation and DNA damage.

**Figure 1:**
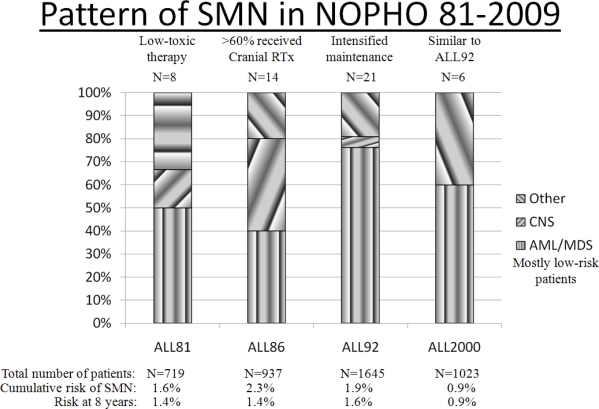
Pattern and incidence of second neoplasms (SMN) among Nordic patients treated during four consecutive Nordic protocol periods. Numbers at the top of columns are the total number of SMNs in that protocol period. CNS = central nervous system; Rtx = radiotherapy; AML = acute myeloid leukemia; MDS = myelodysplaisa.

Based on these findings, the predominance of therapy-related acute myeloid leukemia and myelodysplasia (t-AML/MDS) in the two most recent Nordic ALL protocols (Figure 1), and since 75% of the SMNs occurred among standard risk patients, we analyzed in depth the occurrence of SMN in the NOPHO ALL92 by taking advantage of an extensive registration of individual data on maintenance therapy during the first years of the protocol.

## Patients and Methods:

Patients: From January 1992 until October 2001, 1614 children 1.0–14.9 years of age were diagnosed with B-cell precursor or T-cell ALL in the Nordic countries (Denmark, Finland, Iceland, Norway, and Sweden) and were treated according to the NOPHO ALL92 protocol.^8^

Risk grouping: The risk group criteria are given in table 1.^13^ The patients who had higher risk features at diagnosis and were assigned to the very high risk (VHR) treatment arm received prophylactic or therapeutic central nervous system (CNS) irradiation as well as LSA_2_L_2_ instead of MTX/6MP maintenance therapy.^14^

Induction therapy and consolidation therapy have previously been published in detail.^13^

CNS irradiation and stem cell transplantation: Due to CNS disease at diagnosis or allocation to the very high risk protocol arm 123 non-transplanted patients (7.6% of the total study population) received cranial irradiation in 1^st^ remission (CR1). 31 boys and 21 girls with higher-risk criteria received a hematopoitetic stem cell transplantation in CR1.^15^

Oral 6MP/MTX maintenance therapy was initiated at treatment week 13 (standard risk, SR), 32 (intermediate risk, IR), or 63 (high risk, HR) and scheduled to continue until 2 years (IR and HR) or 2½ years (SR) after diagnosis. Between 1992 and 1996, 538 patients with SR-, IR-, or HR-ALL were included in the randomised ALL-92 maintenance therapy trial.^16^ As part of that study we registered all data on MTX and 6MP doses as well as all blood counts (28.000 data sets in total), and furthermore added data on MTX and 6MP dosing and blood counts during maintenance therapy for the patients with SMN that were not included in the randomised study. The genotype (low activity polymorphisms G460A and A719G) and/or erythrocyte TPMT activity was available for 609 patients.^17^ Erythrocyte TPMT activity was measured during maintenance therapy and at least 8 weeks from the last red blood cell transfusion. For 484 of these patients only the erythrocyte TPMT activity was available. The antimode of the TPMT activity distribution was 14 IU/ml, and patients with TPMT below that level, and for whom no TPMT genotype was available, were classified as TPMT presumed heterozygous.

## Results and Discussion:

With a median follow-up of 10.4 years for patients who remain in first remission, a total of 20 patients developed an SMN at a median of 3.4 years from the diagnosis of ALL (risk at 12 years: 1.6%) (Table 1). The 12-year cumulative risk of developing an SMN (pSMN_12y_) was 1.6 +/− 0.4%. The projected risk of SMN among the non-transplanted patients was significantly related to the risk group and was 2.4 +/− 0.7% for SR-ALL, 1.2 +/− 0.7% for IR-ALL, and 0.3 +/− 0.3% for higher risk ALL patients (p=0.007) (Table 1). The cumulative risk of developing SMN was 1.7 +/− 0.4% for the 1316 patients who were in CR1 when they initiated MTX/6MP maintenance therapy vs 0% for the 169 patients in CR1 at the start of LSA_2_L_2_ maintenance therapy. Twelve of the 20 SMN patients died within 21 months from diagnosis of their SMN with a median time to death of 8.8 months and a projected survival of 39% +/− 11%.

Sixteen of the twenty SMNs were t-AML/MDS, and 9 of these had either monosomy 7 (n=7) or 7q deletions (n=2). Eight of the nine -7/7q- t-AML/MDS cases occurred among patients with high-hyperdiploidy (n=4), t(12;21)(p13;q22) (n=1), or a normal/missing (n=4) karyotype at diagnosis of ALL, which indicates a biological propensity for non-disjunction, although the results did not reach statistical significance. In Cox multivariate analysis, longer duration of oral MTX/6MP maintenance therapy (p=0.02; being longest for standard risk patients) and presence of high-hyperdiploidy (p=0.07) were related to increased risk of SMN. Among all 562 SR-ALL patients, the 161 patients with a high-hyperdiploid karyotype had a 4.8 +/− 1.8% risk of developing SMN compared to a 1.5 +/− 0.6% risk for the remaining 401 SR patients (p=0.02).

The patients who developed an SMN had significantly lower TPMT activity than the remaining patients (Median: 12.1 vs 18.1 IU/ml; p=0.02), and they received 10–15% higher 6MP doses than those that did not develop an SMN, and this was the case both among patients classified as TPMT low activity (Median: 55.5 (N=3) vs 50.3 mg/m^2^ (N=57); p=0.31) and those classified as TPMT wild type (Median: 69.7 (N=9) vs 60.4 mg/m^2^ (N=418); p=0.03). The positive relation between 6MP dosage and risk of SMN is likely to reflect that TPMT-mediated thiopurine methylation, not least through 6-methyl-thioinosine 5′-monophosphate, will inhibit purine de novo synthesis,^18^ and since 6-methyl-thioinosine 5′-monophosphate levels preferentially increase with upward dose adjustments of 6MP,^19^ this may cause enhanced DNA-6TGN incorporation. Such a relationship is supported by the recent demonstration of a positive correlation between erythrocyte levels of methylated 6MP metabolites versus the index between DNA-6-thioguanine nucleotide levels in nucleated cells and the erythrocyte levels of 6-thioguanine nucleotides.^20^

Several drugs have been suggested to modify the risk of SMN by promoting the growth of a malignant clone or by interference with DNA-repair. Such agents include granulocyte colony stimulating factor (),^21^ L-asparaginase,^22^ and thiopurines.^11^,^23^,^24^ The present population-based, retrospective study strongly indicates that maintenance therapy, and especially 6MP pharmacotherapy, influenced the development of SMNs, since 6MP can cause DNA damage,^25^–^27^ and the risk of t-AML/MDS was related to i) longer duration of 6MP/MTX maintenance therapy, ii) low TPMT activity, and iii) significantly higher doses of 6MP for TPMT wild type patients. During oral 6MP therapy 1:10^3^–1:10^4^ DNA nucleotides in leukocytes have been shown to be substituted with 6TGN (DNA-6TGN),^20^ but it is unknown, if the risk of t-AML/MDS is related to the individual leukocyte DNA-6TGN levels.

## Canclusion:

The relation between TPMT activity was not found in a recent large German BFM-study,^28^ but this may reflect both significant differences between that study and the present Nordic study with respect to the MTX/6MP dosages in the two protocols, and furthermore by the fact that only genotyping was used in the BFM study, whereas most patients in the Nordic study was classified by TPMT phenotyping. Thus, it remains to be explored whether TPMT genotyping or phenotyping is superior in predicting the risk of SMN. Since 2001, determination of TPMT geno-/phenotype has been mandatory in NOPHO protocols, and during the initial maintenance therapy 6MP dose is reduced to 50 mg/m^2^ for TPMT heterozygous patients. With longer follow-up it will be explored, if this approach has reduced the risk of SMN for such patients without compromising their low relapse rate.^17^

To further increase the biological understanding of the development of SMN, an international study on SMN chaired by Maria Grazia Valsecchi (AIEOP) and Kjeld Schmiegelow (NOPHO) on behalf of the Ponte di Legno group has been initiated. This stuy will collect data on more than 700 SMNs occuring as the first event in children treated for ALL by 17 major childhood ALL groups in U.S., Europe and Asia.

## Figures and Tables

**Table 1.** Risk group, therapy, and second malignant neoplasms in NOPHO ALL-92 protocol

	SR-ALL	IR-ALL	HR-ALL	VHR-ALL

Risk criteria	Age: 2.0–9.9 and WBC: <10x10^9^/L No HR/VHR-criteria	Age: 1.0–1.9 or ≥10.0 and/or WBC: 10–49 x10^9^/L. No HR/VHR-criteria	WBC≥50x10^9^/L, T-cell, mediastinal or CNS or testicular or lymphomatous disease, t(4;11) or t(9;22), M3 d15 or M2/3 d29, no VHR-criteria	HR-ALL at diagnosis & age ≥5y & i) CNS leukemia, or ii) lymphomatous leukemia, or iii) d15 M3 or a d29 M2/3 BM, or iv) T-ALL with ≥1 other HR-features
Patients	562	590	239	223
Doxorubicin/daunomycin^1^	120	240	280	400
Cyclophosphamide^1^	0	3.000	3.000	6.600
Cranial irradiation	No	No	No	Yes
6MP/MTX (weeks)	117	72	41	0–8
Dead in CR1/relapse/SMN	7/85/12	9/106/4	8/80/2	10/64/2
pEFS/pOS	0.81/0.90	0.79/0.90	0.62/0.74	0.66/0.74
AML/MDS/solid tumor	5/7/0	1/1/1	2*/0/1	0/0/1*
12 year pSMN^2^	2.4 +/− 0.7%	1.2 +/− 0.7%	1.2 +/− 0.8% in total 0.3 +/− 0.8 % for non-transplanted	

AML = acute myeloid leukemia; CNS = central nervous system; Dead in CR1 = dead in first remission; M2/3 BM = ≥5%/25% leukemic blasts in bone-marrow; MDS=myelodysplastic syndrome; 6MP/MTX = duration of oral 6-mercaptopurine/methotrexate maintenance therapy in weeks; pEFS/pOS = the 12-year probability of event-free survival and overall survival, respectively; SR/IR/HR/VHR = standard/intermediate/high/very high risk acute lymphoblastic leukemia; WBC = white blood cell count;

1 Cumulative dose in mg/m^2^;

2 The 12-year probability of developing a second malignant neoplasm;

* Three of the four second malignant neoplasms (SMN) occurred after stem cell transplantation in 1^st^ remission.
